# Supplementary figures and images for: Muscle stem cells in Duchenne muscular dystrophy exhibit molecular impairments and altered cell fate trajectories impacting regenerative capacity
Source: Cell Death Dis. 2025 Jun 5;16(1):437. doi: 10.1038/s41419-025-07755-1 (PMC12141486; doi:10.1038/s41419-025-07755-1)

Uncropped blots for Figure S7D

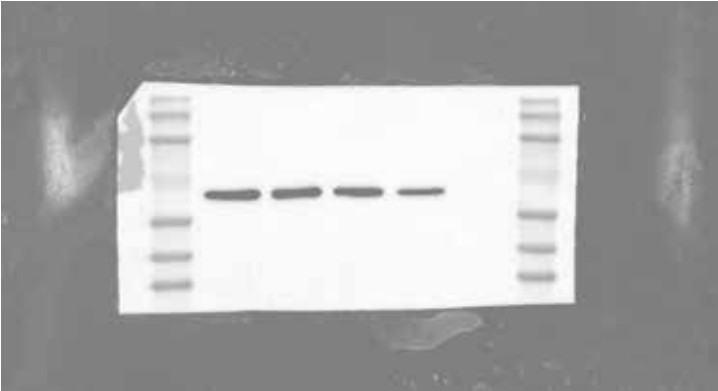

p62

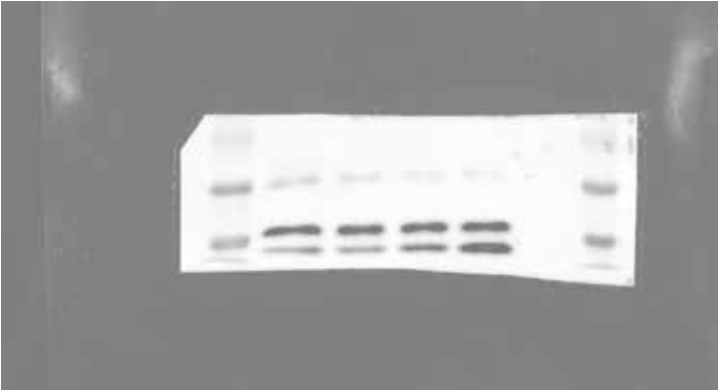

LC3B

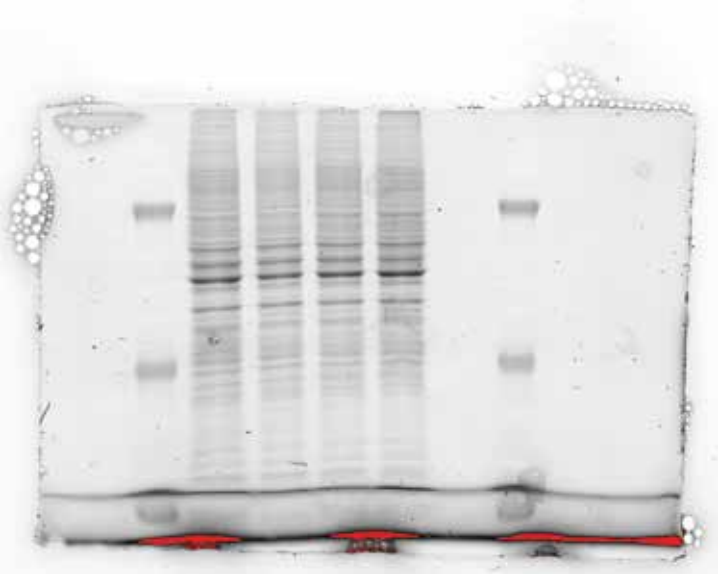

Total  
Protein  
(TCE)

Supplement: Supplementary file 2 — Source Western blots [file 41419_2025_7755_MOESM2_ESM.pdf]
